# An Activity-Based Proteomics with Two-Dimensional Polyacrylamide Gel Electrophoresis (2D-PAGE) for Identifying Target Proteases in *Arabidopsis* Apoplastic Fluid

**DOI:** 10.21769/BioProtoc.5226

**Published:** 2025-03-05

**Authors:** Sayaka Matsui, Yoshikatsu Matsubayashi

**Affiliations:** Division of Biological Science, Graduate School of Science, Nagoya University, Chikusa, Nagoya, Japan

**Keywords:** Extracellular protease, *Arabidopsis*, Native two-dimensional electrophoresis, Fluorescence-quenching peptide substrate, Mass spectrometry, Proteomics

## Abstract

Plant proteases participate in a wide variety of biological processes, including development, growth, and defense. To date, numerous proteases have been functionally identified through genetic studies. However, redundancy among certain proteases can obscure their roles, as single-gene loss-of-function mutants often exhibit no discernible phenotype, limiting identification through genetic approaches. Here, we describe an efficient system for the identification of target proteases that cleave specific substrates in the *Arabidopsis* apoplastic fluid. The method involves using *Arabidopsis*-submerged culture medium, which contains apoplastic proteases, followed by native two-dimensional electrophoresis. Gel fractionation and an in-gel peptide cleavage assay with a fluorescence-quenching peptide substrate are then used to detect specific proteolytic activity. The active fraction is then subjected to mass spectrometry–based proteomics to identify the protease of interest. This method allows for the efficient and comprehensive identification of proteases with specific substrate cleavage activities in the apoplast.

Key features

• Targets *Arabidopsis thaliana* secreted protease but may be applicable to other plant species and intracellular proteases if protease-enriched samples are available.

• The protocol involves an in-gel peptide cleavage assay of native two-dimensional gels diced with SAINOME plates, using a fluorescence-quenching substrate.

• Facilitates the efficient identification of proteases with the desired activity from the entire sample, without restricting the analysis to a specific class of proteases.

## Graphical overview


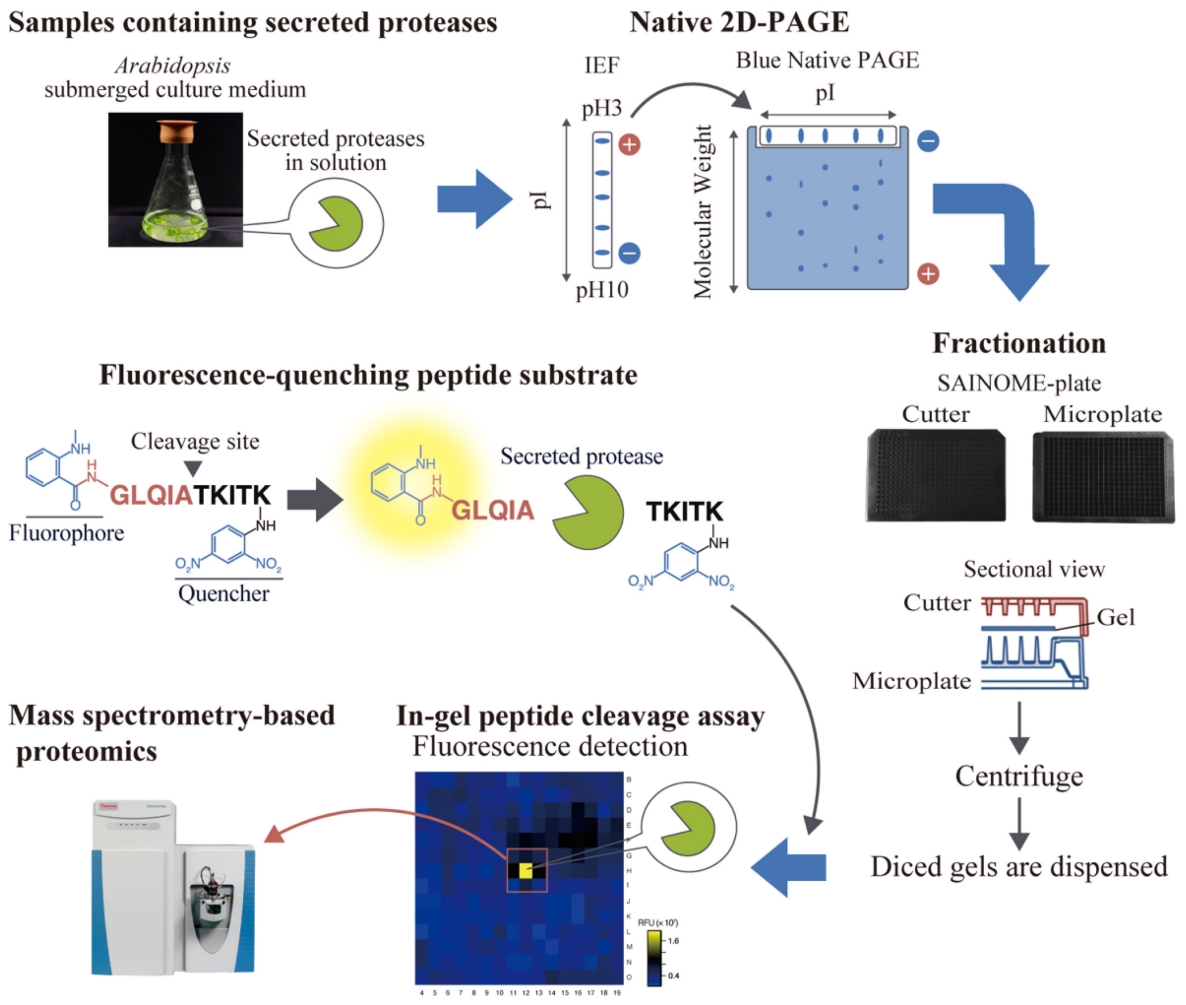


**High-throughput system for identifying proteases that cleave specific substrates in *Arabidopsis* apoplastic fluid.** *Arabidopsis*-submerged culture medium containing secreted proteases is subjected to native two-dimensional electrophoresis. After electrophoresis, the gel is fractionated using a SAINOME plate, and an in-gel peptide cleavage assay is performed using a fluorescence-quenching peptide substrate that fluorescently detects proteolytic activity at the specific cleavage site. Mass spectrometry–based proteomics is performed to identify proteases in the active fraction.

## Background

Plant proteases participate in a variety of physiological processes, including development, growth, and defense [1]. Their roles extend beyond protein degradation to include protein activation and regulation of protein localization through limited and site-specific proteolysis [2]. Among the numerous proteases, secreted proteases in the apoplast have received increasing attention in recent years, mainly because of their roles in immunity and peptide hormone processing [3–5]. In the model plant *Arabidopsis thaliana*, more than 800 putative protease genes have been identified, nearly 300 of which encode secreted proteases with signal peptides [1,6,7].

Proteases often exhibit functional redundancy, which complicates their study through genetic approaches. To address this issue, some studies have employed inhibitors that specifically target particular protease families and suppress their function [8–11]. Alternatively, strategies focusing on changes in transcript levels or enzyme activity under specific conditions have been used to identify proteases of interest [12,13]. Protease identification has also been attempted by narrowing down candidate proteases based on the similarity between the cleavage site of a target substrate and the cleavage sequence of a known protease [14,15]. However, these approaches are all indirect and do not directly assess the specificity of the recognition sequence of the enzyme. Consequently, these methods are not always effective for identifying the protease responsible for cleaving a specific substrate protein.

We have developed a high-throughput system for identifying proteases involved in the cleavage of specific substrates at defined cleavage sites from samples containing apoplastic proteases. This approach employs native two-dimensional (2D) electrophoresis, followed by gel dicing using a SAINOME plate and an in-gel peptide cleavage assay with a fluorescence-quenching peptide substrate [16–19]. Using this system, we recently identified secreted proteases involved in the C-terminal cleavage of the immunogenic peptide flg22, derived from the bacterial flagellar protein flagellin [20]. Here, we provide a step-by-step method for identifying secreted proteases that cleave specific substrates at defined sites, using the identification of flagellin-cleaving proteases as an example. This method is broadly applicable to any proteases that retain enzymatic activity following native 2D electrophoresis.

## Materials and reagents

**Biological materials**

1. *Arabidopsis thaliana* accession Col-0 submerged culture medium [20] (see General note 1)

**Reagents**

1. Gamborg's B5 medium salt mixture (Shiotani M.S., catalog number: 399-00621)

2. Sucrose (Wako, catalog number: 199-00027)

3. Nicotinic acid (Nacalai Tesque, catalog number: 24326-52)

4. Pyridoxine hydrochloride (Wako, catalog number: 165-05401)

5. Thiamin hydrochloride (Wako, catalog number: 201-00852)

6. *Myo*-inositol (Sigma-Aldrich, catalog number: I5125)

7. Glycine (Wako, catalog number: 077-00735)

8. Agar (Wako, catalog number: 016-11875)

9. Spectra/Por 7 membrane (molecular weight cutoff 10 kDa) (Repligen, catalog number: 132117)

10. 3-[(3-Cholamidopropyl)dimethylammonio]propanesulfonate (CHAPS) (Dojindo, catalog number: C008)

11. Immobilized pH gradient (IPG) buffer, pH 3–10 NL (GE Healthcare, catalog number: 17-6000-88)

12. (+/-)-Dithiothreitol (DTT) (Wako, catalog number: 049-08972)

13. Bromophenol blue (BPB) (Wako, catalog number: 021-02911)

14. Immobiline DryStrip pH 3–10 NL, 7 cm (IPG strip) (Cytiva, catalog number: 17-6001-12)

15. Immobiline DryStrip cover fluid (Cytiva, catalog number: 17-1335-01)

16. 6-Aminocaproic acid (LKT Laboratories, catalog number: A4935)

17. Glycerol (Nacalai Tesque, catalog number: 17018-25)

18. 2-Amino-2-hydroxymethyl-1,3-propanediol (Tris) (Wako, catalog number: 204-07885)

19. Coomassie brilliant blue G 250 (CBB G-250) (Fluka, catalog number: 27815)

20. NativePAGE running buffer (20×) (Invitrogen, catalog number: BN2001)

21. NativePAGE 4%–16%, Bis-Tris, 1.0 mm, mini protein gels, 15-well (Invitrogen, catalog number: BN1004BOX)

22. NativeMark^TM^ unstained protein standard (Invitrogen, catalog number: LC0725)

23. Nma-flg[47–56]-Dnp [fluorescence-quenching substrate, Nma-GLQIATKITK(Dnp)-NH_2_] (Peptide Institute, custom-made) (see General Note 2)

24. 2-Morpholinoethanesulfonic acid, monohydrate (MES) (Dojindo, catalog number: GB12)

25. Methanol (Wako, catalog number: 131-01826)

26. Acetic acid (Wako, catalog number: 017-00256)

27. Lysyl endopeptidase (Lys-C) (Wako, catalog number: 125-05061)

28. Trypsin (Promega, catalog number: V5111)

**Solutions**

1. B5 vitamin solution (100×) (see Recipes)

2. B5 medium (see Recipes)

3. Rehydration solution A (see Recipes)

4. Rehydration solution B (see Recipes)

5. Rehydration solution (see Recipes)

6. Equilibration solution (see Recipes)

7. Native PAGE anode buffer (see Recipes)

8. Native PAGE cathode buffer (see Recipes)

9. In-gel peptide cleavage assay solution (see Recipes)

10. Fixing solution (see Recipes)

**Recipes**

**1. B5 vitamin solution (100×)**

**Table:** 

Reagent	Final concentration	Quantity or Volume
Nicotinic acid	50 mg/L	25 mg
Pyridoxine hydrochloride	50 mg/L	25 mg
Thiamin hydrochloride	10 mg/L	5 mg
*Myo*-inositol	10 g/L	5 g
Glycine	200 mg/L	100 mg
Milli-Q water	n/a	Up to 500 mL
Total		n/a	500 mL

**2. B5 medium**

**Table:** 

Reagent	Final concentration	Quantity or Volume
Gamborg's B5 medium salt mixture	n/a	One bag (3.3 g)
B5 vitamin solution (100×)	1×	10 mL
Sucrose	1% (w/v)	10 g
Milli-Q water	n/a	Up to 1 L
Total		n/a	1 L

Adjust pH to 5.7 with 1 N KOH. Autoclave at 121 °C for 20 min. For solid medium, add 7 g of agar before autoclaving.

**3. Rehydration solution A**

**Table:** 

Reagent	Final concentration	Quantity or Volume
CHAPS	4% (w/v)	40 mg
IPG buffer	0.5% (v/v)	5 μL
DTT (15.9 mg/mL)	0.62 mg/mL	39 μL
Milli-Q water	n/a	Up to 1 mL
Total		n/a	1 mL

**4. Rehydration solution B**

**Table:** 

Reagent	Final concentration	Quantity or Volume
CHAPS	2% (w/v)	20 mg
IPG buffer	0.5% (v/v)	5 μL
BPB [0.05% (w/v)]	0.002% (w/v)	40 μL
Milli-Q water	n/a	Up to 1 mL
Total	n/a	1 mL

**5. Rehydration solution**

**Table:** 

Reagent	Final concentration	Quantity or Volume
Rehydration solution A	12.5% (v/v)	20 μL
Rehydration solution B	87.5% (v/v)	140 μL
Total	n/a	160 μL

**6. Equilibration solution**

**Table:** 

Reagent	Final concentration	Quantity or Volume
6-Aminocaproic acid	450 mM	0.59 g
Glycerol	25.5% (v/v)	2.55 mL
Tris-HCl (0.5 M, pH 6.8)	50 mM	1 mL
CBB G-250	0.02% (w/v)	2 mg
Milli-Q water	n/a	Up to 10 mL
Total	n/a	10 mL

**7. Native PAGE anode buffer**

**Table:** 

Reagent	Final concentration	Quantity or Volume
20× Native PAGE running buffer	1×	10 mL
Milli-Q water	n/a	Up to 200 mL
Total	n/a	200 mL

**8. Native PAGE cathode buffer**

**Table:** 

Reagent	Final concentration	Quantity or Volume
20× Native PAGE running buffer	1×	5 mL
CBB G-250 [0.4% (w/v)]	0.002% (w/v)	500 μL
Milli-Q water	n/a	Up to 100 mL
Total	n/a	100 mL

**9. In-gel peptide cleavage assay solution**

**Table:** 

Reagent	Final concentration	Quantity or Volume
Nma-flg[47–56]-Dnp (0.4 mM)	40 μM	1.7 mL
MES-KOH (pH5.7)	25 mM	850 μL
Milli-Q water	n/a	14.45 mL
Total	n/a	17 mL

**10. Fixing solution**

**Table:** 

Reagent	Final concentration	Quantity or Volume
Methanol	20% (v/v)	20 mL
Acetic acid	7.5% (v/v)	7.5 mL
Milli-Q water	n/a	72.5 mL
Total	n/a	100 mL

**Laboratory supplies**

1. 300 mL flask (Iwaki, catalog number: 4980FK300)

2. 15 mL centrifuge tubes (Corning, catalog number: 430791)

3. 1.5 mL microtubes (e.g., BMBio, catalog number: BM-15F)

4. Stainless steel surgical blade, No. 14 (FEATHER Safety Razor, model: No. 14)

5. Tweezers (e.g., rubisTech, catalog number: SS-SA)

6. Square Petri dish (Eiken Chemical, catalog number: AW2000)

7. SAINOME, black [SAINOME consists of a microplate (384-well) and a cutter] (Sainome, catalog number: SAI-386-B)

## Equipment

1. Rotary vacuum evaporator (EYELA, model: N-N series)

2. High-speed micro centrifuge CF15RN (Hitachi Koki, model: himac CF15RN)

3. Angle rotor (Hitachi Koki, model: T11A31)

4. Angle rotor (Hitachi Koki, model: T15AP31)

5. Strip holder, 7 cm (Cytiva, catalog number: 80-6416-87)

6. Ettan IPGphor II isoelectric focusing unit (Amersham Biosciences, catalog number: 80-6505-03)

7. Mini-slab electrophoretic apparatus (Bio Craft, model: BE-211)

8. High-speed micro centrifuge CF16RN (Hitachi Koki, model: himac CF16RN)

9. Swing rotor (Koki Holdings, model: T5S32)

10. VICTOR Nivo multimode plate reader equipped with a 340/20 nm excitation filter and a 435/20 nm emission filter (PerkinElmer, catalog number: HH35000500)

11. 340/20 nm filter (PerkinElmer, catalog number: HH35000912)

12. 435/20 nm filter (PerkinElmer, catalog number: HH35000918)

13. Extrahera (Biotage, catalog number: 414001)

14. Mass spectrometer (Thermo Fisher Scientific, model: Q Exactive Hybrid Quadrupole-Orbitrap Mass Spectrometer)

15. Gradient pump (Thermo Fisher Scientific, model: Dionex U3000)

## Software and datasets

1. VICTOR Nivo software ver. 4.0 (PerkinElmer, 2020 May)

2. Proteome Discoverer ver. 2.3 (Thermo Fisher Scientific)

## Procedure

**A. Native 2D electrophoresis**

1. Sample preparation

a. Prepare *Arabidopsis* whole-plant-submerged culture as described previously [20]. Briefly, transplant forty 6-day-old seedlings, germinated on B5 solid medium, into 100 mL of B5 liquid medium in a 300 mL flask. Incubate them for 7–11 days at 22 °C under continuous light without shaking.

*Note: The flask should not be continuously shaken, but it is recommended to gently shake it by hand a few times a day.*

b. Collect the spent medium by decantation (*Arabidopsis-*submerged culture medium).

c. Concentrate 100 mL of the *Arabidopsis*-submerged culture medium to 10 mL using a rotary evaporator with the water bath set to 37 °C.

d. Transfer the concentrated sample into 15 mL centrifuge tubes and centrifuge at 11,900*× g* for 5 min using a himac CF15RN (T11A31 rotor).

e. Dialyze the supernatant overnight with stirring at 4 °C against 3 L of reverse osmosis (RO) water using a Spectra/Por 7 membrane.

f. Transfer the dialyzed sample to 15 mL centrifuge tubes and centrifuge at 11,900*× g* for 5 min using a himac CF15RN (T11A31 rotor).

g. Transfer the supernatant to a new 15 mL centrifuge tube, freeze at -80 °C, and lyophilize.

h. Dissolve the lyophilized powder in 125 μL of rehydration solution and transfer to a 1.5 mL tube.

2. Native isoelectric focusing (IEF)

a. Centrifuge 125 μL of the dehydration solution containing the sample at 18,800*× g* for 5 min using a himac CF15RN (T15AP31 rotor).

b. Load the supernatant into a 7 cm strip holder.

c. Place the immobilized pH gradient (IPG strip) in the strip holder with the gel-side down in the dehydration solution.

d. Apply DryStrip cover fluid to the strip and cover the strip holder with the plastic lid.

e. Place the strip holder on the Ettan IPGphor platform and perform IEF using the following program:

Rehydrate for 14 h at 20 °C.

Perform IEF at 50 μA/strip and 20 °C using the following steps:

Step 1: 500 V for 30 min

Step 2: 1,000 V for 30 min

Step 3: 5,000 V for 100 min

*Note: The maximum voltage may not be reached during the program. The rehydration time can be adjusted for convenience but must be longer than 10 h.*

**Figure 1.**
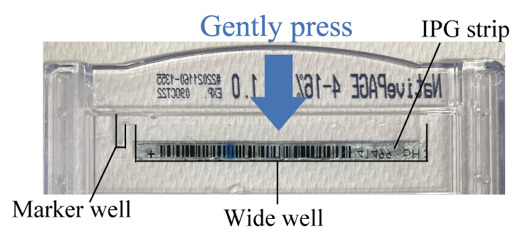
Preparation for native PAGE. Native PAGE gel with an IPG strip placed on top.

3. Blue native polyacrylamide gel electrophoresis (PAGE)

a. After IEF, place the IPG strip in 10 mL of equilibration solution in a 15 mL tube.

b. Equilibrate the IPG strip at 4 °C for 15 min.

c. Rinse the IPG strip with native PAGE anode buffer.

d. Remove wells from the native PAGE gel using a surgical blade and tweezers to prepare a gel with one protein marker well and one wide well.

e. Using tweezers, place the IPG strip in the wide well and gently press the strip onto the top surface of the native PAGE gel (Figure 1).

*Note: Slightly trim the ends of the strip to fit into the well. Avoid air bubbles between the strip and the gel.*

f. Place the gel into the electrophoretic apparatus and pour native PAGE cathode buffer into the upper buffer tank and native PAGE anode buffer into the lower buffer tank.

g. Load 3 μL of NativeMark unstained protein standard into the protein marker well.

h. Run electrophoresis at 150 V for approximately 90 min or until the dye front line reaches the end of the gel.

**B. In-gel peptide cleavage assay**

**Figure 2.**
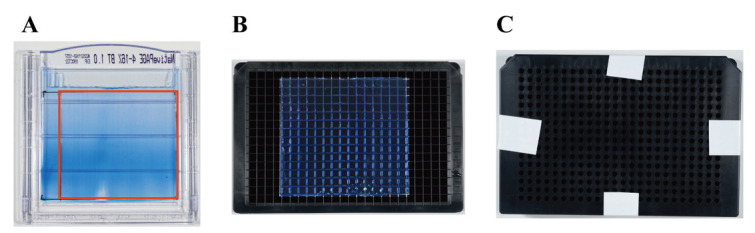
Fractionation of the gel using the SAINOME plate. A. Gel after native two-dimensional electrophoresis. Remove all parts of the gel except the part enclosed by the red square. B. Placement of the gel on the SAINOME plate. C. SAINOME plate with cutter positioned and secured with tape.

1. Fractionation of the gel after electrophoresis using a SAINOME plate

a. Remove the top and bottom of the gel with a surgical blade, taking care to remove the contact surface with the IPG strip and the area below the dye front. Also, remove the marker lane (Figure 2A).

*Note: The marker lane can be used as a reference for evaluating native PAGE separation and may be stained or destained as appropriate.*

b. Rinse the gel with 25 mM MES-KOH pH 5.7 in a square Petri dish.

c. Place the gel on a SAINOME 384-well microplate (Figure 2B).

d. Position the SAINOME cutter, which is designed to fit on top of the plate, on the gel.

e. Press the cutter firmly onto the gel and secure the plate and cutter together with tape (Figure 2C).

f. Centrifuge the assembly at 2,000*× g* for 5 min using a himac CF16RN (T5S32 rotor).

*Note: By centrifugation, the gel is cut into 4.5 mm squares and dispensed into the 384-well plate.*

**Figure 3.**
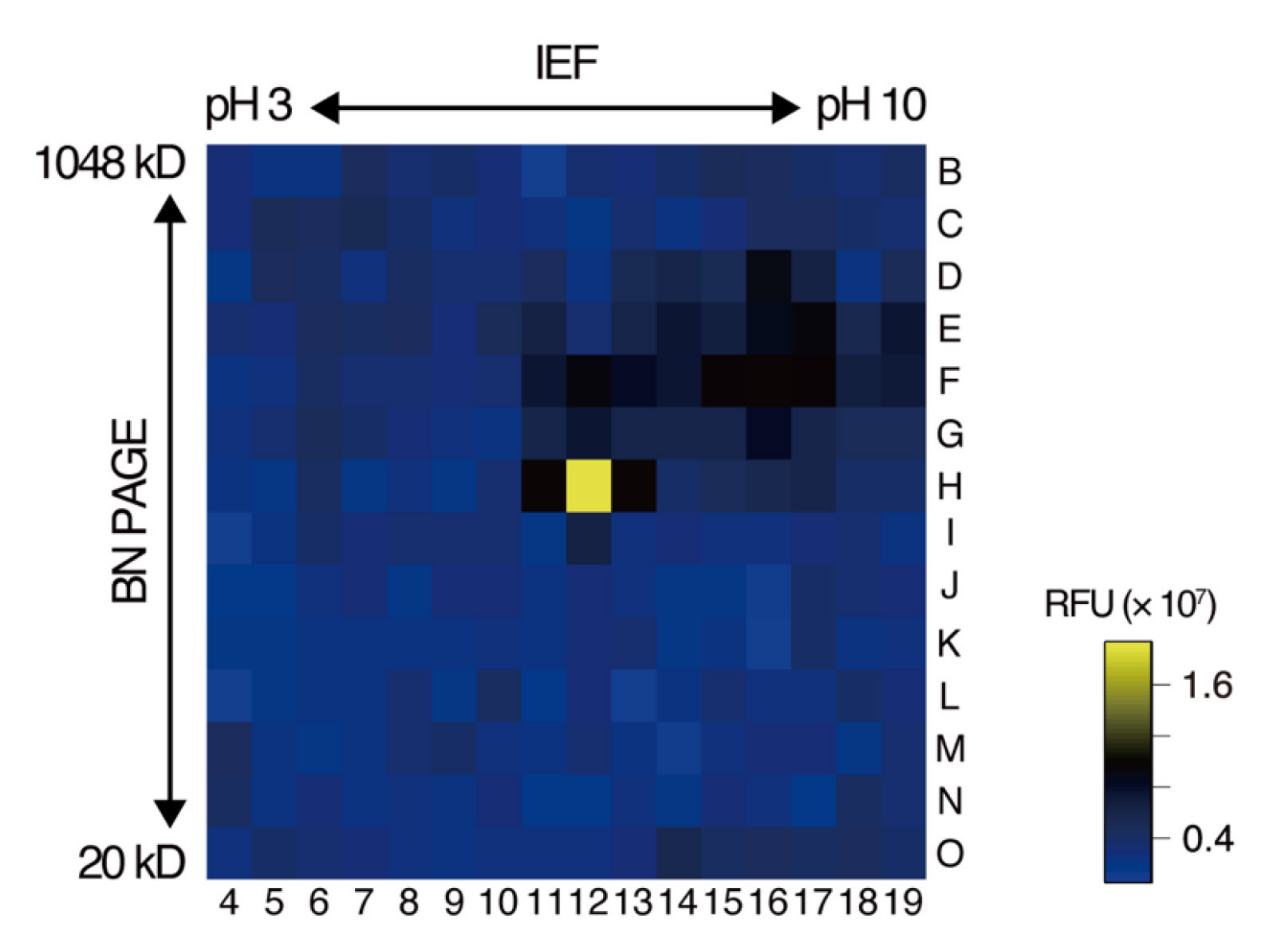
Representative results of the in-gel peptide cleavage assay using a fluorescence-quenching substrate. Fluorescence intensity 16 h after substrate addition is shown as an example. The numbers and letters represent the columns and rows, respectively, of the microplate used in the assay. Fluorescence intensity is color-coded according to the scale shown on the right. BN PAGE: blue native polyacrylamide gel electrophoresis; IEF: isoelectric focusing; RFU: relative fluorescence units.

2. In-gel peptide cleavage assay of fluorescence-quenching substrate

a. Remove the cutter from the plate and add 60 μL of in-gel peptide cleavage assay solution to each well containing gel pieces.

b. Measure the fluorescence intensity in each well using a plate reader with an excitation wavelength of 340 nm and an emission wavelength of 435 nm every hour for 16 h (Figure 3).

**C. Proteomic analysis of the active gel fractions**

1. In-gel protein digestion

a. Collect assay solution and gel fragments separately from the fluorescence-positive and control fluorescence-negative wells and place each into separate 1.5 mL tubes.

*Note: Nano-liquid chromatography–mass spectrometry (nano-LC–MS) analysis of the collected assay solution can confirm whether correct cleavage of the substrate occurred.*

b. Fix the gel fragments with fixing solution at room temperature for 1 h.

c. Transfer the fixed gel fragments to a 96-well filter plate with tweezers and perform reduction and alkylation using Extrahera, as described previously [21].

d. Transfer the gel fragments with tweezers to a new 1.5 mL tube and digest with Lys-C/trypsin as previously described [21].

2. Nano-liquid chromatography–tandem mass spectrometry (nano-LC–MS/MS) analysis

a. Analyze 7.25 μL aliquots by nano-LC–MS/MS using a system combining a Q Exactive Hybrid Quadrupole-Orbitrap mass spectrometer with a Dionex U3000 gradient pump as described previously [21].

3. MS/MS data analysis

a. Process and analyze the MS/MS raw files with Proteome Discoverer 2.3 using the SEQUEST HT algorithm, searching against the TAIR10 *Arabidopsis* protein database.

b. Use the following search parameters: peptide mass range, 350–5,000 Da; enzyme specificity, trypsin (Full), with up to two missed cleavages; minimum peptide length, six residues; maximum equal modifications per peptide, 3; precursor ion and peptide fragment mass tolerances, ±10 ppm and ±0.02 Da, respectively; static modification, carbamidomethyl (Cys); and dynamic modifications, oxidation (Met).

c. Extract proteases with signal peptides from the resulting high-confidence master protein list using TAIR Gene Ontology (GO) and SignalP 6.0 [7].

d. Identify secreted proteases specifically present in the active fraction based on peptide-spectrum match (PSM) and abundance scores.

## Validation of protocol

This protocol or parts of it has been used and validated in the following research article:

• Matsui et al. [20]. *Arabidopsis* SBT5.2 and SBT1.7 subtilases mediate C-terminal cleavage of flg22 epitope from bacterial flagellin. *Nature Communications* (Figure 3, panels a and b).

## General notes and troubleshooting

**General notes**

1. Biological material: Although *Arabidopsis-*submerged culture medium is used as an example, this method is not limited to *Arabidopsis* samples. Any sample in which proteases retain activity after native 2D electrophoresis can be used.

2. Fluorescence-quenching peptide substrate: Nma-flgN[47–56]-Dnp, with a purity of 97.4%, specifically detects proteolytic activity at the C-terminus of the flg22 domain [20]. A paired fluorophore and quencher are introduced at opposite ends of the peptide substrate. Hydrolysis of an internal peptide bond increases the distance between the donor and acceptor, preventing quenching of the fluorophore by the quencher and enhancing the fluorescence signal. The proteolytic activity of other proteases can also be detected by altering the amino acid sequence of the peptide substrate.
